# Translation, cultural adaptation, and validation of the German EMPATHIC-30G questionnaire for use in the neonatal intensive care setting

**DOI:** 10.3389/fped.2025.1650141

**Published:** 2025-10-03

**Authors:** Katja Odermatt, Marie Roumet, Christine Arnold, Juerg Burren, Jos M. Latour, Mark T. Marston, Martin Nagl-Cupal, Ralph C. A. Rippe, Christoph E. Schwarz, Alexander Simma, Hannah Ferentzi, André Kidszun

**Affiliations:** ^1^ Division of Neonatology, Department of Pediatrics, Inselspital, Bern University Hospital, University of Bern, Bern, Switzerland; ^2^ Department of Clinical Research, University of Bern, Bern, Switzerland; ^3^ Department of Intensive Care and Neonatology, and Children’s Research Center, University Children’s Hospital Zurich, Zurich, Switzerland; ^4^ Faculty of Health, School of Nursing and Midwifery, University of Plymouth, Plymouth, United Kingdom; ^5^ Neonatal and Pediatric Intensive Care Units, Children’s University Hospital Basel, Basel, Switzerland; ^6^ Department of Nursing Science, University of Vienna, Vienna, Austria; ^7^ Institute of Education and Child Studies, Leiden University, Leiden, Netherlands; ^8^ Clinic of Neonatology, Center for Pediatric and Adolescent Medicine, University of Heidelberg, Heidelberg, Germany; ^9^ Department of Pediatric Cardiology, Pulmonology and Pediatric Intensive Care Medicine, University Children’s Hospital Tübingen, Tübingen, Germany; ^10^ Department of Congenital Heart Disease – Pediatric Cardiology, Developmental Pediatrics, Deutsches Herzzentrum der Charité, Berlin, Germany; ^11^ Charité – Universitätsmedizin Berlin, corporate member of Freie Universität Berlin and Humboldt-Universität zu Berlin, Berlin, Germany

**Keywords:** EMPATHIC-30, neonatal intensive care unit, parent satisfaction, psychometric evaluation, neonatology, reliability, validity, quality of care

## Abstract

**Background:**

Parental satisfaction is a key quality indicator in neonatal intensive care units (NICUs). While the EMPATHIC-30 questionnaire is widely used internationally, no validated German version exists for NICU settings. This study aimed to translate, culturally adapt, and validate the EMPATHIC-30G for use in German-speaking NICUs.

**Methods:**

A structured Delphi process involving multidisciplinary experts and parents guided the translation and cultural adaptation, including forward-backward translation and cognitive debriefing. The German version was validated in a prospective observational study at a Swiss NICU. Parents of infants hospitalized for ≥24 h completed the questionnaire at discharge.

**Results:**

A total of 228 questionnaires were completed (138 by mothers, 89 by fathers, 1 by another caregiver). Exploratory factor analysis identified six latent factors for mothers and four for fathers, explaining ∼70% of variance. Internal consistency was excellent (McDonald's omega/Cronbach's alpha: 0.97/0.96 for mothers, 0.98/0.97 for fathers). Construct validity was supported by moderate to strong correlations with global satisfaction indicators; discriminant validity was confirmed by low correlation with unrelated variables. At the domain level, ceiling effects exceeded the 15% threshold across all five domains, though inter-item correlations remained acceptable. Several items showed substantial non-response due to being marked “not applicable” reflecting variability in clinical experiences.

**Conclusion:**

The EMPATHIC-30G is a reliable and valid instrument for assessing parent satisfaction in German-speaking NICUs. However, ceiling effects may limit its sensitivity in high-satisfaction settings. Further evaluation in multicenter samples is recommended.

## Introduction

The admission of an infant to a neonatal intensive care unit (NICU) is a profoundly stressful and often traumatic experience for parents. Fear, helplessness, and emotional distress are commonly reported, particularly due to concerns about the infant's survival, health outcomes, and the highly technical environment of the NICU (1–3). The approach of family-centered care (FCC) plays a pivotal role in this vulnerable period. Respect, information sharing, participation and collaboration are the core principles of FCC, ensuring active involvement in decision-making and equal collaboration between families and healthcare providers. FCC leads to improved parent satisfaction, stress reduction and enhanced communication (4, 5). Measuring parent satisfaction is therefore essential not only as a component of FCC but also as a key indicator of quality of care (6, 7). Several instruments have been developed to assess parent satisfaction in neonatal settings, including the Neonatal Satisfaction Survey (NSS-8), the Neonatal Index of Parent Satisfaction (NIPS), and the Empowerment of Parents in The Intensive Care (EMPATHIC) questionnaire (8–10). Among these, the EMPATHIC instruments have been most widely used and validated across pediatric and neonatal settings in various countries (11–15). The EMPATHIC questionnaire plays a key-role in advancing FCC. By systematically capturing parents' satisfaction and experiences in NICU settings, it provides data to evaluate and improve implementation of FCC in clinical practice.

The original EMPATHIC was developed in the Netherlands for pediatric intensive care units (PICUs). Since then, it was shortened to the 30-item EMPATHIC-30 version, which has demonstrated robust psychometric properties and is increasingly favored for its feasibility in clinical settings (16). The EMPATHIC-30 was tailored to pediatric and neonatal intensive care settings, particularly NICUs, PICUs (Pediatric Intensive Care Units) and PCICUs (Pediatric Cardiac Intensive Care Units) to allow comparison across clinical populations (17, 18). Despite its international use, the EMPATHIC-30 has not yet been validated for NICU settings in German. Moreover, validated data comparing mothers' and fathers' perceptions of satisfaction with NICU care are lacking.

Standardized tools to measure parent satisfaction are essential for internal quality improvement, international benchmarking, and collaborative research. In neonatal critical care, a validated German version of the EMPATHIC-30 (EMPATHIC-30G) would facilitate the implementation of FCC in German-speaking NICUs and enable cross-cultural comparisons of family experiences, thereby supporting quality assurance and improved health outcomes after NICU discharge (19, 20).

This study aimed to (1) translate and culturally adapt the EMPATHIC-30 for use in German-speaking pediatric settings and (2) evaluate its psychometric properties in a real-world NICU context.

## Methods

### Study design and setting

This prospective observational validation study was conducted at the NICU of the University Hospital Bern (Inselspital), Switzerland, with participant recruitment from May 2023 to November 2024.

### Translation and cultural adaptation

The translation and cultural adaptation of the EMPATHIC-30G instrument followed international guidelines for patient-reported outcome measures, including recommendations from ISPOR by Wild et al. (21).

A multinational expert working group—including neonatologists, pediatric cardiologists, nursing scientists, psychologists, and a statistician from Germany, Austria, the United Kingdom, the Netherlands, and Switzerland—was convened. A structured Delphi process was used to achieve consensus on culturally appropriate terminology for use in Germany, Austria, and Switzerland. The adaptation process included:

1. forward translation of the original Dutch version by two independent bilingual translators;
2. reconciliation and back-translation by a third translator unfamiliar with the original;
3. expert panel review to resolve discrepancies and ensure conceptual and experiential equivalence; and
4. cognitive debriefing with parents from various target settings to assess item clarity and relevance.

Minor revisions were made based on parental feedback, aiming to preserve fidelity to the original while enhancing clarity and contextual fit for German-speaking NICUs. The final EMPATHIC-30G is included in the Supplementary Material.

### Participants and data collection

All parents (mothers, fathers, or other caregivers) of infants admitted to the NICU for ≥24 h were eligible. Inclusion criteria were age ≥18 years and self-reported sufficient German language proficiency. Parents of infants who died during hospitalization were excluded. At NICU discharge, eligible parents received a QR code linking to the electronic version of the questionnaire hosted in the REDCap database, accessible only after providing written informed consent. Sociodemographic data were collected for both parents (e.g., age, country of origin, education) and infants (e.g., birth weight, length of NICU stay).

### Instrument description, scoring and handling of missing data

The original EMPATHIC-30 is a standardized parent-report measure of satisfaction with family-centered care in pediatric and neonatal intensive care. It includes 30 items covering five domains:

• Information
• Care and Treatment
• Organization
• Parental Participation
• Professional Attitude

Each item is rated on a 6-point Likert scale from 1 (“certainly not”) to 6 (“certainly yes”) with an additional option of “not applicable” to account for items that did not pertain to the respondent's experience. Additionally, four items assess overall satisfaction on a 10-point scale (global satisfaction indicators):

1. We would recommend this department to anyone in a similar situation.
2. If we were ever in this situation again, we would like to return to this department.
3. How do you rate our physicians' team overall?
4. How do you rate our nursing team overall?

The overall EMPATHIC score was calculated as the mean of all 30 satisfaction items. Dimension scores were calculated as the mean of the items within each respective domain. Scores were computed only if >75% of the relevant items were completed (i.e., not missing or marked “not applicable”). For the total score, a minimum of 23 answered items was required. For dimensions containing 5, 6, or 8 items, a minimum of 4, 5, and 6 valid responses, respectively, was required to compute the score.

### Statistical analysis

We aimed to collect data from at least 150 participants, exceeding the minimum sample size of 50 recommended in published guidelines for scale validation (22). Descriptive statistics were used to summarize sociodemographic characteristics and response distributions. All analyses were conducted separately for mothers and fathers using R version 4.4.2.

To explore the underlying factor structure of the EMPATHIC-30G, we performed an Exploratory Factor Analysis (EFA) using the principal-factor method applied to the item correlation matrix. Orthogonal varimax rotation was employed to improve interpretability, and the number of factors retained was determined using the Kaiser criterion (eigenvalues >1). Factor loadings were then examined to evaluate the alignment of individual items with the intended domains.

Psychometric properties of the EMPATHIC-30G were assessed across several dimensions. Acceptability was evaluated based on the proportion of missing or “not applicable” responses at the item level, as well as floor and ceiling effects at the domain level. Missing data rates below 5% and floor or ceiling effects below 15% (i.e., the proportion of respondents selecting the lowest or highest possible rating) were considered acceptable. Internal consistency was measured using McDonald's omega and Cronbach's alpha for each domain and the overall score, with values ≥0.70 regarded as satisfactory. Item-level analysis included item-total correlations (target >0.20) and average inter-item correlations (target >0.30) (23). Construct validity was assessed using Spearman's rank correlations between the EMPATHIC-30G total and domain scores and four global satisfaction indicators. Discriminant validity was evaluated by testing for correlations with unrelated variables, such as the season of birth, which were hypothesized to be unassociated with satisfaction scores.

### Ethics

This study was approved by the Ethics Commission of the Canton of Berne (Req-2022-00886), Switzerland, which declared that the study did not fall under the provisions of Article 2, Paragraph 1 of the Swiss Federal Law on Human Research. All participants provided written informed consent prior to enrolment.

## Results

### Participant characteristics

Data collection was completed on March 31st, 2025. A total of 228 questionnaires were included in the analysis: 138 completed by mothers, 89 by fathers, and 1 by a respondent identifying as “other”. The latter was included in overall descriptive statistics but excluded from subgroup analyses due to low frequency.

The mean age was 33.7 years (SD = 4.5) for mothers and 35.0 years (SD = 5.3) for fathers. Most participants were born in Switzerland (85% of mothers and 88% of fathers). Educational levels were comparable between groups: 41% of mothers and 40% of fathers held a university degree, followed by completed apprenticeships (33% in both groups). Sociodemographic characteristics of parents are presented in Table 1.

**Table 1:** Parents’ characteristics.

Characteristic	Mother (*N* = 138)	Father (*N* = 89)	Other (*N* = 1)
Age (years)			
Mean (SD)	33.7 (4.5)	35.0 (5.3)	37.0 (NA)
Missing	3	2	0
Country of birth, *n* (%)			
Switzerland	115 (85%)	77 (88%)	1 (100%)
Other	21 (15%)	11 (13%)	0 (0%)
Missing	2	1	0
After school education, *n* (%)			
None	0 (0%)	1 (1.1%)	0 (0%)
Apprenticeship	46 (33%)	29 (33%)	1 (100%)
Technical and vocational education and training	34 (25%)	23 (26%)	0 (0%)
University	56 (41%)	36 (40%)	0 (0%)
Other	2 (1.4%)	0 (0%)	0 (0%)

A total of 162 infants were represented. Of these, 18% (*n* = 29) had a birth weight below 1,500 g. The median length of NICU stay was 10 days [interquartile range (IQR): 6–25]. Most infants (94%, *n* = 149) were discharged home, while 6.3% (*n* = 10) were transferred to another ward or institution. Infant characteristics are summarized in Table 2.

**Table 2:** Infants’ characteristics.

Characteristic	Overall (*n* = 162)
Length of stay in hospital (days)	
Median [IQR]	10 [6, 25]
Missing	8
Infant weight at birth, *n* (%)	
<1,500 g	29 (18%)
1,500 g and more	130 (82%)
Missing	3
Discharge place, *n* (%)	
Home	149 (94%)
Another ward/institution	10 (6.3%)
Missing	3

### EMPATHIC-30G scores

The median total EMPATHIC-30G score was nearly identical between groups, at 5.65 (IQR: 5.28–5.86) for mothers and 5.66 (IQR: 5.39–5.86) for fathers. Domain-specific median scores ranged from 5.60 (Information and Organization) to 5.83 (Professional Attitude) in both groups. Full details on median scores and interquartile ranges are presented in Table 3.

**Table 3:** EMPATHIC-30G scores—median [IQR] and mean (SD).

Domain	Mother (*N* = 138)	Father (*N* = 89)
Information		
Median [Q1, Q3]	5.60 [5.00, 6.00]	5.60 [5.00, 5.80]
Mean (SD)	5.36 (0.80)	5.35 (0.84)
Missing	6	3
Care & Treatment		
Median [Q1, Q3]	5.73 [5.20, 5.88]	5.63 [5.25, 5.88]
Mean (SD)	5.43 (0.77)	5.42 (0.78)
Missing	6	6
Organization		
Median [Q1, Q3]	5.60 [5.00, 5.80]	5.60 [5.00, 6.00]
Mean (SD)	5.34 (0.73)	5.37 (0.76)
Missing	8	6
Parental participation		
Median [Q1, Q3]	5.67 [5.20, 6.00]	5.73 [5.50, 6.00]
Mean (SD)	5.44 (0.81)	5.57 (0.77)
Missing	12	7
Professional attitude		
Median [Q1, Q3]	5.83 [5.67, 6.00]	5.83 [5.67, 6.00]
Mean (SD)	5.67 (0.72)	5.70 (0.77)
Missing	5	5
EMPATHIC-30G total score		
Median [Q1, Q3]	5.65 [5.28, 5.86]	5.66 [5.39, 5.86]
Mean (SD)	5.45 (0.68)	5.50 (0.72)
Missing	6	5

### Exploratory factor analysis

An exploratory factor analysis (EFA) was performed separately for mothers and fathers using the principal-factor method with varimax rotation. For mothers, six factors were retained, accounting for approximately 70% of the total variance. For fathers, four factors were retained, explaining 72.7% of the total variance. In both groups, the first factor accounted for a substantial portion of the variance—31.6% for mothers and 46.4% for fathers. In contrast, the variance explained by the subsequent factors was below 10% for mothers and below 14% for fathers (see Supplementary Table S1).

Although the EMPATHIC-30 was originally developed to reflect a five-domain structure, our analysis did not replicate this model. Instead, the dominance of the first factor suggests a predominantly unidimensional structure in our sample.

In both groups, items from the Professional Attitude domain showed the most consistent loading, with nearly all clustering strongly on the first factor. Items from the Information and Parental Participation domains also demonstrated coherent loading patterns, particularly in the paternal subsample. However, cross-loadings were observed, especially for items in the Organization and Care and Treatment domains, which loaded across multiple factors in both groups. For mothers, item groupings were generally less distinct than for fathers, although the overall structure remained comparable.

Despite differences in the number and configuration of retained factors between groups, a consistent pattern emerged: items that loaded together for fathers tended to group similarly in the maternal sample. Detailed factor loadings are provided in the Supplementary Table S2.

### Acceptability

The EMPATHIC-30G demonstrated good overall acceptability. The proportion of non-response (including missing and “not applicable” responses) for the total score was low—4.3% for mothers and 5.6% for fathers. No floor effects were observed, and ceiling effects remained within acceptable limits for the total score, at 8.3% for mothers and 7.1% for fathers.

At the domain level, acceptability was more limited. The proportion of missing data approached or exceeded the commonly accepted 5% threshold in several domains. For mothers, missing data ranged from 3.6% (Professional Attitude) to 8.7% (Parental Participation); for fathers, from 3.4% (Information) to 7.9% (Parental Participation). Ceiling effects exceeded the 15% threshold across all five domains for both groups (see Table 4).

**Table 4:** Non-response rates, floor and ceiling effects on domain level.

Score	Mothers				Fathers			
	*N*	Floor effect	Ceiling effect	Missing	*N*	Floor effect	Ceiling effect	Missing
Information	132	1 (0.8%)	40 (30.3%)	6 (4.3%)	86	0 (0.0%)	21 (24.4%)	3 (3.4%)
Care & Treatment	132	0	30 (22.7%)	6 (4.3%)	83	0	20 (24.1%)	6 (6.7%)
Organization	130	0	29 (22.3%)	8 (5.8%)	83	0	23 (27.7%)	6 (6.7%)
Parental Participation	126	0	45 (35.7%)	12 (8.7%)	82	0	29 (35.4%)	7 (7.9%)
Professional Attitude	133	1 (0.8%)	64 (48.1%)	5 (3.6%)	84	1 (1.2%)	40 (47.6%)	5 (5.6%)
**EMPATHIC-30G total score**	**132**	**0**	**11 (8.3%)**	**6 (4.3%)**	**84**	**0**	**6 (7.1%)**	**5 (5.6%)**

Bold indicates EMPATHIC-30 full-scale scores comprising all questionnaire items.

At the item level, four items showed non-response rates greater than 10% in both groups. These included:

• Q3: Information about the effects of medication (mothers: 17.4%, fathers: 15.7%)
• Q7: Prevention and treatment of pain (mothers: 14.5%, fathers: 15.7%)
• Q15: Telephone availability (mothers: 10.9%, fathers: 19.1%)
• Q21: Ability to stay close during intensive care procedures (mothers: 34.8%, fathers: 37.1%)

The response option “not applicable” accounted for a large portion of these missing values, ranging from 9.4% (Q15; mothers) to 35% (Q21; fathers). A detailed overview of missing and “not applicable” responses per item is provided in the Supplementary Table S3.

Ceiling effects were also observed at the item level, exceeding the acceptable threshold in all 30 items. Rates ranged from 27.6% (Q17: There was enough space around our child's bed; mothers) to 91.8% (Q9: Our child's comfort was taken into account by nurses; fathers).

### Internal consistency

The EMPATHIC-30G demonstrated excellent internal consistency. McDonald's omega for the total score was 0.97 (95% CI: 0.96–0.98) for mothers and 0.98 (95% CI: 0.97–0.99) for fathers, indicating high reliability. At the domain level, omega values ranged from 0.78 (Organization) to 0.92 (Professional Attitude) for mothers, and from 0.76 (Organization) to 0.95 (Professional Attitude) for fathers, reflecting good to excellent internal consistency across all subscales. Cronbach's alpha values for the total score and domains were in a similar range. The average inter-item correlation for the total score was also within the acceptable range, at 0.48 for mothers and 0.57 for fathers. Detailed results for McDonald's omega, Cronbach's alpha, and inter-item correlations are presented in Table 5. Further information regarding item-total correlations and dropped Cronbach's alpha for all items can be found in Supplementary Table S4.

**Table 5:** Mcdonalds omega, Cronbach's alpha and average inter-item correlation.

Mothers				Fathers			
EMPATHIC Score (number of items per dimension)	McDonald's omega^a^	Cronbach's alpha^a^	Average inter-item correlation	EMPATHIC Score (number of items per dimension)	McDonald's omega^a^	Cronbach's alpha^a^	Average inter-item correlation
Information (5)	0.82 [0.77, 0.87]	0.81 [0.76, 0.86]	0.53	Information (5)	0.83 [0.76, 0.89]	0.82 [0.75, 0.87]	0.55
Care & Treatment (8)	0.89 [0.85, 0.92]	0.87 [0.84, 0.90]	0.53	Care & Treatment (8)	0.90 [0.85, 0.93]	0.89 [0.86, 0.92]	0.57
Organization (5)	0.78 [0.70, 0.83]	0.75 [0.68, 0.81]	0.41	Organization (5)	0.76 [0.66, 0.84]	0.76 [0.67, 0.83]	0.45
Parental Participation (6)	0.88 [0.84, 0.92]	0.85 [0.80, 0.88]	0.51	Parental Participation (6)	0.93 [0.90, 0.96]	0.92 [0.89, 0.94]	0.63
Professional Attitude (6)	0.92 []0.89, 0.94	0.90 [0.87, 0.93]	0.63	Professional Attitude (6)	0.95 [0.93, 0.96]	0.95 [0.93, 0.96]	0.75
**EMPATHIC-30G total score** (30)	**0.97 [0.96, 0.98]**	**0.96 [0.95, 0.97]**	**0.48**	**EMPATHIC-30G total score** (30)	**0.98 [0.97, 0.99]**	**0.97 [0.96, 0.98]**	**0.57**

Bold indicates EMPATHIC-30 full-scale scores comprising all questionnaire items.

^a^ McDonald's Omega and Cronbach's alpha are presented with the associated 95% Feldt confidence intervals.

### Construct validity

Construct validity was supported by moderate to strong correlations between EMPATHIC-30G domain and total scores and the four global satisfaction indicators. For mothers, correlations with the total score ranged from 0.48 (Willingness to return to the unit) to 0.69 (Perceived physician performance). For fathers, correlations ranged from 0.35 (Perceived nursing performance) to 0.74 (Perceived physician performance). On the domain level, Spearman's rank correlation coefficients for mothers ranged from 0.33 (Information ↔ Nursing performance) to 0.69 (Care and Treatment ↔ Physician performance); for fathers, from 0.12 (Information ↔ Nursing performance) to 0.68 (Care and Treatment ↔ Physician performance). Correlation values for all domains and indicators are shown in Table 6.

**Table 6:** Spearman correlations with the four validity indicators.

Domain	Mothers				Fathers			
	Would you recommend the neonatal unit to others? (0–6 scale)	Would you return to the unit in the future if needed? (0–6 scale)	Rate of physicians’ performance (1–10 scale)	Rate of nurses’ performance (1–10 scale)	Would you recommend the neonatal unit to others? (0–6 scale)	Would you return to the unit in the future if needed? (0–6 scale)	Rate of physicians’ performance (1–10 scale)	Rate of nurses’ performance (1–10 scale)
Information	0.39 [0.23, 0.52]	0.36 [0.20, 0.50]	0.60 [0.48, 0.70]	0.33 [0.17, 0.47]	0.16 [−0.06, 0.36]	0.24 [0.03, 0.43]	0.52 [0.35, 0.66]	0.12 [−0.09, 0.33]
Care & Treatment	0.41 [0.26, 0.55]	0.38 [0.23, 0.52]	0.69 [0.58, 0.77]	0.51 [0.37, 0.62]	0.44 [0.25, 0.60]	0.46 [0.27, 0.62]	0.68 [0.54, 0.78]	0.33 [0.12, 0.51]
Organization	0.49 [0.35, 0.61]	0.46 [0.31, 0.58]	0.40 [0.24, 0.54]	0.46 [0.31, 0.59]	0.33 [0.12, 0.51]	0.37 [0.17, 0.54]	0.36 [0.16, 0.54]	0.19 [−0.03, 0.39]
Parental Participation	0.48 [0.33, 0.60]	0.44 [0.28, 0.57]	0.49 [0.35, 0.61]	0.49 [0.34, 0.61]	0.42 [0.22, 0.58]	0.47 [0.28, 0.62]	0.64 [0.49, 0.75]	0.39 [0.19, 0.56]
Professional Attitude	0.54 [0.41, 0.65]	0.51 [0.38, 0.63]	0.58 [0.46, 0.69]	0.45 [0.30, 0.57]	0.52 [0.34, 0.66]	0.55 [0.38, 0.68]	0.59 [0.43, 0.72]	0.46 [0.27, 0.61]
EMPATHIC-30G total score	0.51 [0.38, 0.63]	0.48 [0.33, 0.60]	0.69 [0.59, 0.77]	0.54 [0.40, 0.65]	0.42 [0.22, 0.58]	0.48 [0.29, 0.63]	0.74 [0.62, 0.82]	0.35 [0.15, 0.53]

Spearman correlations are presented with the associated 95% confidence interval.

### Discriminant validity

Discriminant validity was supported by low correlations between EMPATHIC-30G total or domain scores and the unrelated variable “season of birth”.

## Discussion

The aim of this study was to translate, culturally adapt, and validate the EMPATHIC-30G questionnaire for use in the neonatal intensive care setting. To accomplish this, we combined a rigorous translation and cultural adaption procedure with psychometric testing in a real-world NICU population. The results provide strong support for the reliability, validity, and general feasibility of the German version in a real-world NICU environment.

Exploratory factor analysis (EFA) showed that a large part of the variance in the EMPATHIC-30G was explained by the first component alone—31.6% for mothers and 46.4% for fathers—supporting a predominantly unidimensional structure. The overall high satisfaction levels in our sample, cultural factors, and the standardized nature of NICU care may have reduced variation across domains, leading to stronger loadings on a single factor. The specific characteristics of our parent population (predominantly Swiss-born, highly educated, and from a single center) may also have contributed to the emergence of a unidimensional structure. For both groups, items from the Professional Attitude domain loaded most consistently onto a single factor. In contrast, items from the Organization and Care and Treatment domains were distributed across multiple factors, suggesting conceptual overlap or different interpretations depending on parental perspective. Despite these deviations, clustering patterns were broadly consistent between mothers and fathers. Both our study and Girch et al. were unable to confirm the original five-domain structure of the EMPATHIC questionnaire. While a dominant general factor was observed, Girch et al. also reported poor fit for both a three-factor and a purely unidimensional model, indicating that the factor structure may be more complex and warrants further refinement (18).

Acceptability of the instrument was good, with low overall non-response rates. Only a few items exceeded the 10% threshold, primarily those related to specific clinical procedures (e.g., drug effects, pain management) or practical issues such as telephone availability. High rates of “not applicable” responses suggest that these situations may not have been experienced by all parents (e.g., not having attempted to call the NICU). Rather than reflecting poor item quality, these findings point to clinical variability and may indicate areas for clarification or contextual adaptation in future versions.

Although ceiling effects for the overall score were within acceptable limits, domain- and item-level analyses revealed ceiling effects well above the recommended 15% threshold across all measures. Items with high non-response and pronounced ceiling effects raise concerns about their ability to capture meaningful variation in parental experiences, potentially limiting the instrument's sensitivity in high-performing settings.

Internal consistency was excellent, with McDonald's omega and Cronbach's alpha values exceeding 0.9 for the total score, and good to excellent coefficients across all domains. These results are consistent with prior validations of the EMPATHIC-30 in other languages and cultural contexts. In our study, Cronbach's alpha ranged from 0.75 to 0.95 across domains, comparable to or exceeding values reported by Girch et al. (2022; 0.73–0.85), Lake et al. (2020; 0.68–0.81), Zhuang et al. (2022; 0.67–0.95), and Latour et al. (2013; 0.73–0.81). As in previous studies, the lowest internal consistency was observed in the domain Organization, and the highest in Professional Attitude (also reported by Girch and Zhuang), although Lake found the highest Cronbach's alpha in Parental Participation and Latour in Care and Treatment (16, 18, 24, 25).

Construct validity was supported by moderate to strong correlations between domain scores and global satisfaction indicators. As in Lake et al., perceived physician performance showed the strongest correlation with the overall EMPATHIC-30G score. Notably, lower correlations were observed in the Information domain and for three of four global indicators among fathers, suggesting potential gender differences in information needs or in how information is communicated. This discrepancy merits further qualitative investigation. Global satisfaction items appear to be of particular importance, as they capture parents’ overall impression of care and may serve as intuitive benchmarks complementing the domain-specific scores.

In addition to construct validity, discriminant validity was supported by the absence of associations between EMPATHIC-30G scores and an unrelated variable (season of birth), which was chosen as it is plausibly independent of parental satisfaction with care.

### Limitations

This study has several limitations. First, it was conducted in a single Swiss NICU, which may limit generalizability. Further validation in NICUs across Germany, Austria, and Switzerland is needed. Second, the sample lacked diversity, with most participants being Swiss-born and relatively few fathers included. Third, only parents of surviving infants were included, excluding perspectives of bereaved families.

Future research should include multicenter studies with more diverse samples to confirm the factor structure and test measurement invariance. Longitudinal designs could explore how satisfaction changes during and after hospitalization. Associations with clinical outcomes, staff experience, and organizational factors may help position the EMPATHIC-30G as a broader quality indicator. Qualitative research could clarify how parents interpret items with high ceiling effects or non-response. Finally, digital implementation should be explored to support real-time, family-centered care.

## Conclusion

Overall, our findings reinforce the international relevance of the EMPATHIC-30 and contribute to its broader applicability by validating a German version for use in neonatal intensive care. The EMPATHIC-30G is an acceptable, reliable, and valid instrument for assessing parent satisfaction in NICUs. Its implementation may support quality improvement efforts, enhance family-centered care, and enable benchmarking across German-speaking neonatal units.

## Data Availability

The raw data supporting the conclusions of this article will be made available by the authors, without undue reservation.
