# Supplementary material for: Translation, cultural adaptation, and validation of the German EMPATHIC-30G questionnaire for use in the neonatal intensive care setting
Source: Front Pediatr. 2025 Oct 3;13:1650141. doi: 10.3389/fped.2025.1650141 (PMC12531056; doi:10.3389/fped.2025.1650141)
Supplement: Supplementary file 1 [file Table1.docx]

Supplementary Material

# Supplementary Tables

## Supplementary Table S1 Factor analysis

|  | **Mothers** | | | | | | **Fathers** | | | |
| --- | --- | --- | --- | --- | --- | --- | --- | --- | --- | --- |
|  | **MR1** | **MR5** | **MR3** | **MR2** | **MR6** | **MR4** | **MR1** | **MR4** | **MR3** | **MR2** |
| **Eigenvalues** | 15.17 | 1.73 | 1.45 | 1.02 | 0.90 | 0.86 | 18.31 | 1.82 | 0.88 | 0.79 |
| **Variance (%)** | 31.57 | 9.34 | 9.15 | 7.49 | 7.23 | 5.63 | 46.43 | 13.11 | 7.37 | 5.75 |
| **Cumulative Variance (%)** | 31.57 | 40.91 | 50.06 | 57.56 | 64.79 | 70.42 | 46.43 | 59.53 | 66.91 | 72.65 |

## Supplementary Table S2 Factor analysis – rotated factor loadings

| **Variable** | **Mothers** | | | | | | **Fathers** | | | |
| --- | --- | --- | --- | --- | --- | --- | --- | --- | --- | --- |
|  | **MR1** | **MR5** | **MR3** | **MR2** | **MR6** | **MR4** | **MR1** | **MR4** | **MR3** | **MR2** |
| **Information** | | | | | | | | | | |
| Q_1 | 0.62 | 0.19 | -0.01 | 0.29 | 0.25 | 0.17 | 0.65 | 0.32 | 0.08 | 0.40 |
| Q_2 | 0.68 | 0.18 | 0.05 | 0.24 | 0.27 | 0.15 | 0.82 | 0.28 | 0.15 | 0.30 |
| Q_3 | 0.65 | 0.18 | 0.19 | 0.28 | 0.19 | 0.06 | 0.63 | 0.34 | 0.21 | 0.12 |
| Q_4 | 0.22 | 0.01 | 0.14 | 0.82 | 0.14 | 0.03 | 0.09 | 0.06 | 0.31 | 0.92 |
| Q_5 | 0.72 | 0.01 | 0.45 | 0.11 | -0.02 | 0.07 | 0.74 | 0.24 | -0.02 | 0.27 |
| **Care and Treatment** | | | | | | | | | | |
| Q_6 | 0.39 | 0.51 | 0.20 | 0.20 | 0.20 | 0.33 | 0.69 | 0.45 | 0.15 | 0.16 |
| Q_7 | 0.83 | 0.17 | 0.31 | 0.10 | 0.34 | 0.12 | 0.88 | 0.25 | 0.25 | 0.08 |
| Q_8 | 0.60 | 0.24 | -0.05 | 0.37 | 0.07 | 0.34 | 0.42 | 0.33 | 0.43 | 0.28 |
| Q_9 | 0.85 | 0.24 | 0.23 | -0.01 | 0.20 | 0.09 | 0.93 | 0.27 | 0.10 | 0.06 |
| Q_10 | 0.06 | 0.30 | 0.01 | 0.80 | 0.15 | 0.11 | 0.02 | 0.31 | 0.51 | 0.46 |
| Q_11 | 0.33 | 0.48 | 0.44 | 0.13 | 0.19 | 0.15 | 0.67 | 0.57 | 0.04 | 0.06 |
| Q_12 | 0.31 | 0.70 | 0.17 | 0.28 | 0.14 | 0.00 | 0.26 | 0.71 | 0.33 | 0.12 |
| Q_13 | 0.31 | 0.78 | 0.26 | 0.11 | 0.21 | 0.05 | 0.61 | 0.50 | 0.14 | 0.10 |
| **Organization** | | | | | | | | | | |
| Q_14 | 0.23 | 0.24 | 0.73 | 0.09 | 0.32 | 0.15 | 0.89 | 0.14 | 0.18 | 0.03 |
| Q_15 | 0.48 | -0.03 | 0.39 | 0.08 | 0.15 | 0.39 | 0.70 | 0.62 | 0.02 | 0.12 |
| Q_16 | 0.19 | 0.16 | 0.30 | 0.12 | 0.47 | 0.02 | 0.44 | 0.12 | 0.70 | 0.04 |
| Q_17 | 0.11 | 0.08 | 0.05 | 0.18 | 0.65 | 0.03 | -0.01 | 0.05 | 0.39 | 0.10 |
| Q_18 | 0.48 | 0.27 | 0.13 | 0.00 | 0.67 | 0.25 | 0.65 | 0.44 | 0.35 | 0.08 |
| **Parental Participation** | | | | | | | | | | |
| Q_19 | 0.69 | 0.19 | -0.07 | 0.21 | 0.05 | 0.21 | 0.60 | 0.44 | 0.15 | 0.03 |
| Q_20 | 0.62 | 0.38 | 0.04 | -0.08 | 0.19 | 0.24 | 0.65 | 0.36 | -0.05 | 0.06 |
| Q_21 | 0.27 | 0.11 | 0.21 | 0.12 | 0.10 | 0.92 | 0.83 | 0.35 | 0.11 | 0.15 |
| Q_22 | 0.51 | 0.26 | 0.16 | 0.20 | 0.15 | 0.04 | 0.40 | 0.53 | 0.27 | 0.15 |
| Q_23 | 0.60 | 0.15 | 0.20 | 0.33 | 0.15 | 0.29 | 0.68 | 0.40 | 0.26 | 0.15 |
| Q_24 | 0.74 | 0.20 | 0.39 | 0.11 | 0.16 | 0.11 | 0.85 | 0.35 | 0.17 | -0.04 |
| **Professional Attitude** | | | | | | | | | | |
| Q_25 | 0.74 | 0.36 | 0.25 | 0.01 | 0.09 | 0.12 | 0.80 | 0.31 | 0.12 | 0.19 |
| Q_26 | 0.24 | 0.23 | 0.77 | 0.05 | 0.06 | 0.10 | 0.86 | 0.12 | 0.24 | -0.01 |
| Q_27 | 0.57 | 0.22 | 0.23 | 0.10 | 0.46 | 0.14 | 0.81 | 0.19 | 0.35 | -0.05 |
| Q_28 | 0.82 | 0.21 | 0.33 | -0.01 | 0.22 | 0.08 | 0.87 | 0.33 | 0.08 | 0.16 |
| Q_29 | 0.63 | 0.31 | 0.21 | 0.33 | 0.12 | 0.14 | 0.63 | 0.14 | 0.37 | 0.14 |
| Q_30 | 0.87 | 0.13 | 0.29 | 0.03 | 0.18 | 0.05 | 0.90 | 0.25 | 0.18 | -0.02 |

## Supplementary Table S3 Non-applicable and missing data per item

|  | **Mother** | **Father** |
| --- | --- | --- |
|  | **N = 138^1^** | **N = 89^1^** |
| **Q_1** | | |
| Answered | 133 (96%) | 86 (97%) |
| Not applicable | 3 (2.2%) | 1 (1.1%) |
| Missing | 2 (1.4%) | 2 (2.2%) |
| **Q_2** | | |
| Answered | 134 (97%) | 83 (93%) |
| Not applicable | 2 (1.4%) | 3 (3.4%) |
| Missing | 2 (1.4%) | 3 (3.4%) |
| **Q_3** | | |
| Answered | 114 (83%) | 75 (84%) |
| Not applicable | 20 (14%) | 11 (12%) |
| Missing | 4 (2.9%) | 3 (3.4%) |
| **Q_4** | | |
| Answered | 133 (96%) | 85 (96%) |
| Not applicable | 1 (0.7%) | 2 (2.2%) |
| Missing | 4 (2.9%) | 2 (2.2%) |
| **Q_5** | | |
| Answered | 133 (96%) | 85 (96%) |
| Not applicable | 3 (2.2%) | 2 (2.2%) |
| Missing | 2 (1.4%) | 2 (2.2%) |
| **Q_6** | | |
| Answered | 129 (93%) | 83 (93%) |
| Not applicable | 6 (4.3%) | 3 (3.4%) |
| Missing | 3 (2.2%) | 3 (3.4%) |
| **Q_7** | | |
| Answered | 118 (86%) | 75 (84%) |
| Not applicable | 15 (11%) | 12 (13%) |
| Missing | 5 (3.6%) | 2 (2.2%) |
| **Q_8** | | |
| Answered | 131 (95%) | 83 (93%) |
| Not applicable | 4 (2.9%) | 3 (3.4%) |
| Missing | 3 (2.2%) | 3 (3.4%) |
| **Q_9** | | |
| Answered | 134 (97%) | 85 (96%) |
| Not applicable | 1 (0.7%) | 2 (2.2%) |
| Missing | 3 (2.2%) | 2 (2.2%) |
| **Q_10** | | |
| Answered | 134 (97%) | 78 (88%) |
| Not applicable | 2 (1.4%) | 6 (6.7%) |
| Missing | 2 (1.4%) | 5 (5.6%) |
| **Q_11** | | |
| Answered | 134 (97%) | 84 (94%) |
| Not applicable | 1 (0.7%) | 3 (3.4%) |
| Missing | 3 (2.2%) | 2 (2.2%) |
| **Q_12** | | |
| Answered | 134 (97%) | 83 (93%) |
| Not applicable | 2 (1.4%) | 4 (4.5%) |
| Missing | 2 (1.4%) | 2 (2.2%) |
| **Q_13** | | |
| Answered | 131 (95%) | 83 (93%) |
| Not applicable | 3 (2.2%) | 4 (4.5%) |
| Missing | 4 (2.9%) | 2 (2.2%) |
| **Q_14** | | |
| Answered | 135 (98%) | 85 (96%) |
| Not applicable | 1 (0.7%) | 2 (2.2%) |
| Missing | 2 (1.4%) | 2 (2.2%) |
| **Q_15** | | |
| Answered | 123 (89%) | 72 (81%) |
| Not applicable | 13 (9.4%) | 15 (17%) |
| Missing | 2 (1.4%) | 2 (2.2%) |
| **Q_16** | | |
| Answered | 133 (96%) | 85 (96%) |
| Not applicable | 2 (1.4%) | 2 (2.2%) |
| Missing | 3 (2.2%) | 2 (2.2%) |
| **Q_17** | | |
| Answered | 134 (97%) | 86 (97%) |
| Not applicable | 1 (0.7%) | 1 (1.1%) |
| Missing | 3 (2.2%) | 2 (2.2%) |
| **Q_18** | | |
| Answered | 129 (93%) | 83 (93%) |
| Not applicable | 7 (5.1%) | 4 (4.5%) |
| Missing | 2 (1.4%) | 2 (2.2%) |
| **Q_19** | | |
| Answered | 132 (96%) | 83 (93%) |
| Not applicable | 4 (2.9%) | 4 (4.5%) |
| Missing | 2 (1.4%) | 2 (2.2%) |
| **Q_20** | | |
| Answered | 130 (94%) | 85 (96%) |
| Not applicable | 5 (3.6%) | 2 (2.2%) |
| Missing | 3 (2.2%) | 2 (2.2%) |
| **Q_21** | | |
| Answered | 90 (65%) | 56 (63%) |
| Not applicable | 43 (31%) | 31 (35%) |
| Missing | 5 (3.6%) | 2 (2.2%) |
| **Q_22** | | |
| Answered | 131 (95%) | 84 (94%) |
| Not applicable | 5 (3.6%) | 3 (3.4%) |
| Missing | 2 (1.4%) | 2 (2.2%) |
| **Q_23** | | |
| Answered | 134 (97%) | 84 (94%) |
| Not applicable | 2 (1.4%) | 2 (2.2%) |
| Missing | 2 (1.4%) | 3 (3.4%) |
| **Q_24** | | |
| Answered | 135 (98%) | 84 (94%) |
| Not applicable | 1 (0.7%) | 2 (2.2%) |
| Missing | 2 (1.4%) | 3 (3.4%) |
| **Q_25** | | |
| Answered | 132 (96%) | 85 (96%) |
| Not applicable | 3 (2.2%) | 2 (2.2%) |
| Missing | 3 (2.2%) | 2 (2.2%) |
| **Q_26** | | |
| Answered | 134 (97%) | 84 (94%) |
| Not applicable | 1 (0.7%) | 3 (3.4%) |
| Missing | 3 (2.2%) | 2 (2.2%) |
| **Q_27** | | |
| Answered | 135 (98%) | 86 (97%) |
| Not applicable | 1 (0.7%) | 1 (1.1%) |
| Missing | 2 (1.4%) | 2 (2.2%) |
| **Q_28** | | |
| Answered | 133 (96%) | 84 (94%) |
| Not applicable | 3 (2.2%) | 3 (3.4%) |
| Missing | 2 (1.4%) | 2 (2.2%) |
| **Q_29** | | |
| Answered | 135 (98%) | 86 (97%) |
| Not applicable | 1 (0.7%) | 1 (1.1%) |
| Missing | 2 (1.4%) | 2 (2.2%) |
| **Q_30** | | |
| Answered | 136 (99%) | 84 (94%) |
| Not applicable | 0 (0%) | 3 (3.4%) |
| Missing | 2 (1.4%) | 2 (2.2%) |

^1^ n (%)

## Supplementary Table S4 Item-total correlation and dropped Cronbach’s alpha

|  | **Item-total correlation** | | **Dropped alpha** | |
| --- | --- | --- | --- | --- |
| **Item** | **Mother** | **Father** | **Mother^1^** | **Father^1^** |
| **Q_1** | 0.73 | 0.78 | 0.954 | 0.968 |
| **Q_2** | 0.76 | 0.89 | 0.954 | 0.968 |
| **Q_3** | 0.75 | 0.76 | 0.954 | 0.968 |
| **Q_4** | 0.54 | 0.42 | 0.957 | 0.973 |
| **Q_5** | 0.68 | 0.76 | 0.955 | 0.968 |
| **Q_6** | 0.73 | 0.83 | 0.954 | 0.968 |
| **Q_7** | 0.88 | 0.91 | 0.953 | 0.967 |
| **Q_8** | 0.72 | 0.69 | 0.954 | 0.969 |
| **Q_9** | 0.83 | 0.91 | 0.954 | 0.968 |
| **Q_10** | 0.52 | 0.45 | 0.959 | 0.972 |
| **Q_11** | 0.69 | 0.81 | 0.955 | 0.968 |
| **Q_12** | 0.68 | 0.66 | 0.955 | 0.969 |
| **Q_13** | 0.70 | 0.78 | 0.954 | 0.968 |
| **Q_14** | 0.64 | 0.84 | 0.955 | 0.968 |
| **Q_15** | 0.61 | 0.84 | 0.955 | 0.968 |
| **Q_16** | 0.52 | 0.65 | 0.956 | 0.969 |
| **Q_17** | 0.45 | 0.23 | 0.958 | 0.974 |
| **Q_18** | 0.75 | 0.85 | 0.954 | 0.968 |
| **Q_19** | 0.68 | 0.74 | 0.955 | 0.969 |
| **Q_20** | 0.69 | 0.69 | 0.955 | 0.969 |
| **Q_21** | 0.56 | 0.86 | 0.955 | 0.967 |
| **Q_22** | 0.66 | 0.69 | 0.955 | 0.969 |
| **Q_23** | 0.76 | 0.84 | 0.954 | 0.968 |
| **Q_24** | 0.83 | 0.87 | 0.954 | 0.968 |
| **Q_25** | 0.80 | 0.87 | 0.954 | 0.968 |
| **Q_26** | 0.55 | 0.82 | 0.956 | 0.968 |
| **Q_27** | 0.78 | 0.84 | 0.954 | 0.968 |
| **Q_28** | 0.83 | 0.90 | 0.954 | 0.968 |
| **Q_29** | 0.81 | 0.72 | 0.954 | 0.969 |
| **Q_30** | 0.82 | 0.88 | 0.954 | 0.968 |

^1^ Cronbach’s alpha coefficient if item is dropped

# EMPATHIC-30G Questionnaire


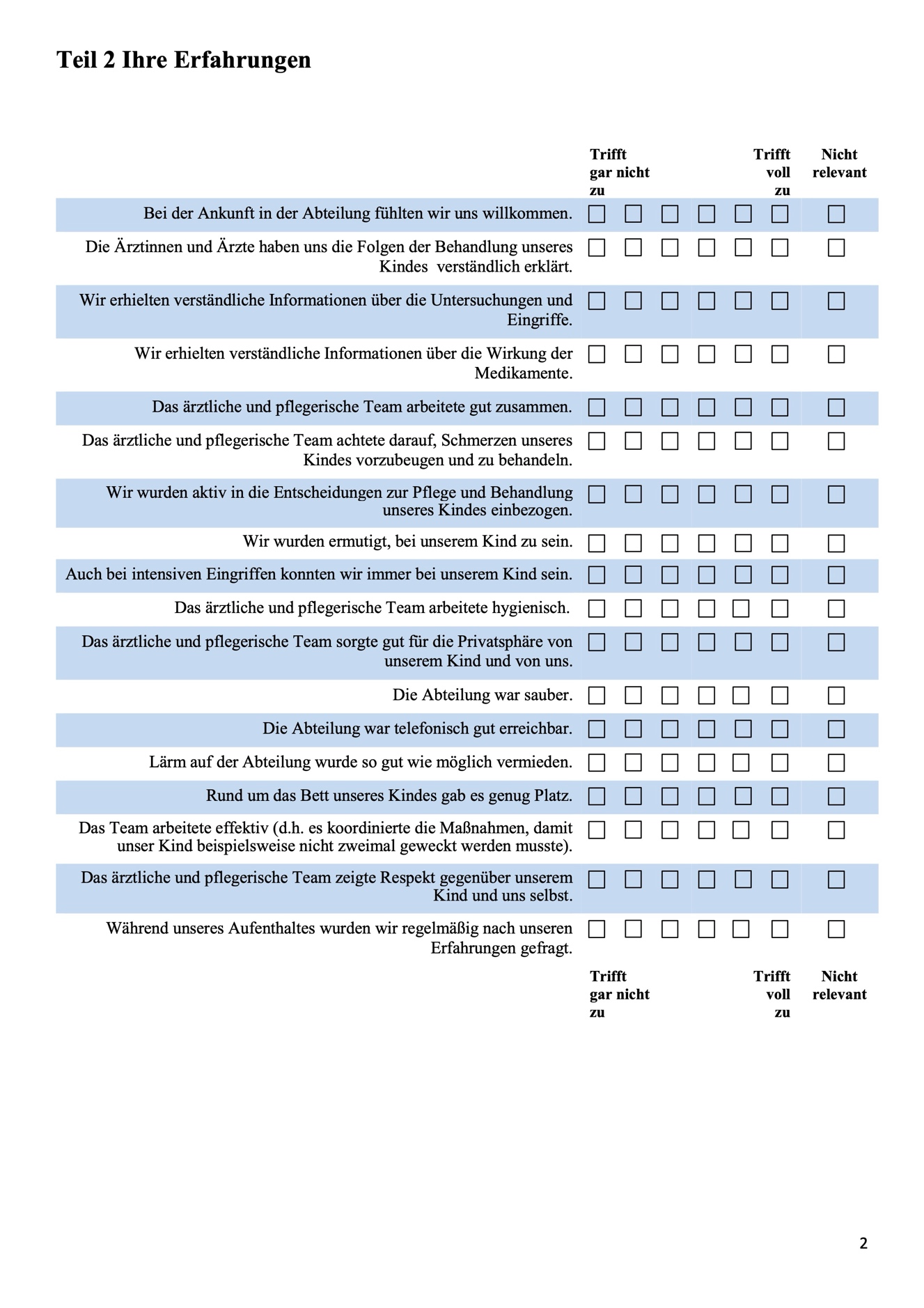


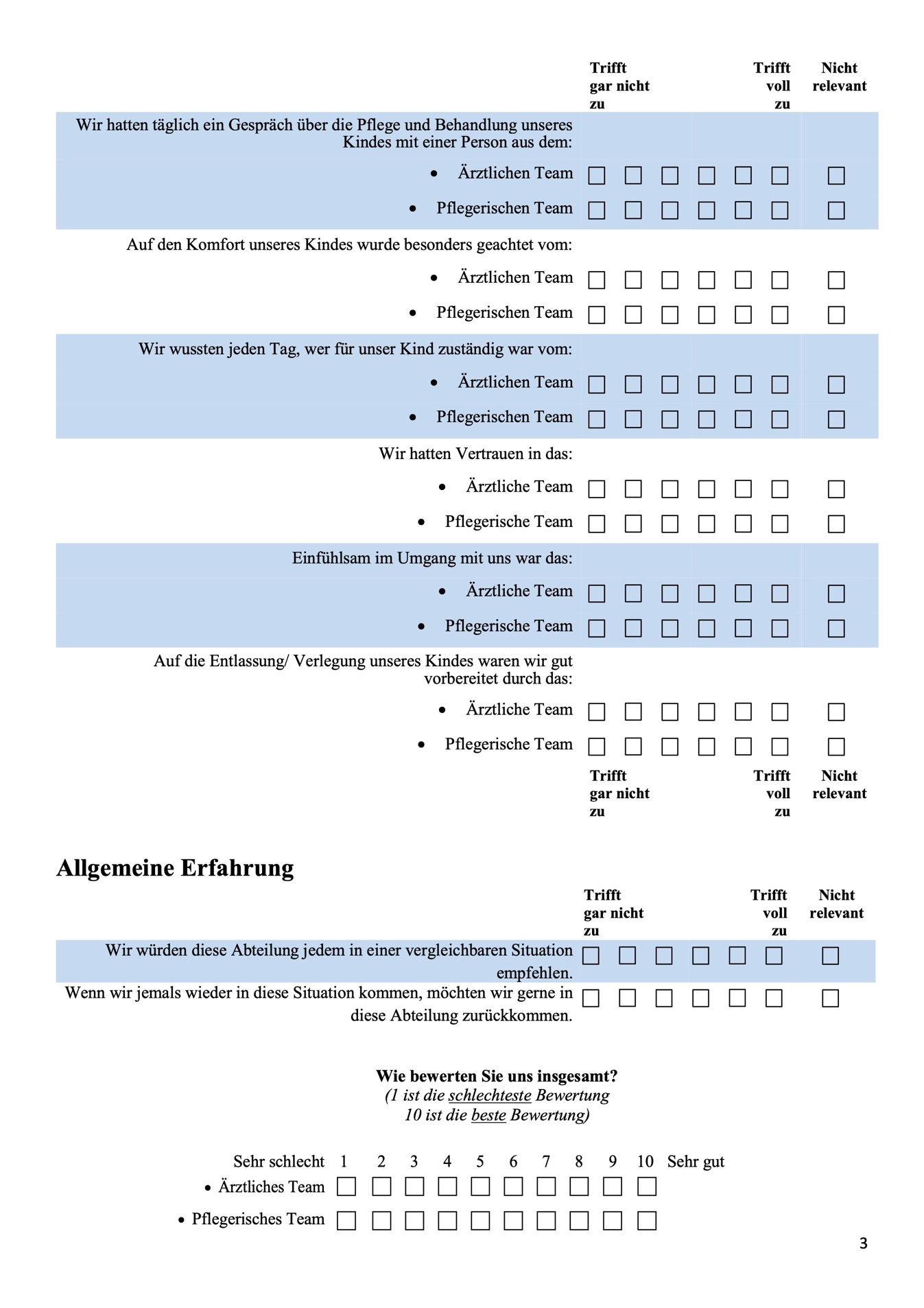


**
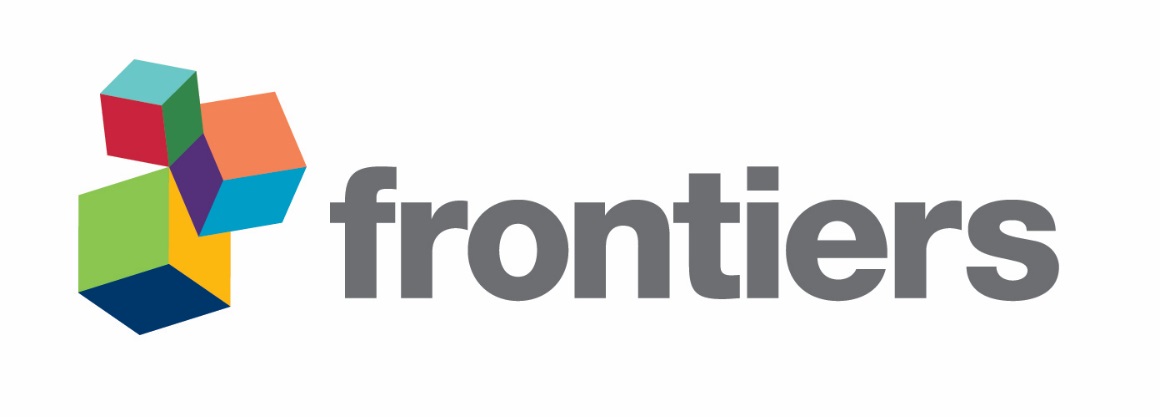
**
